# PRISMA 2020 and PRISMA-S: common questions on tracking records and the flow diagram

**DOI:** 10.5195/jmla.2022.1449

**Published:** 2022-04-01

**Authors:** Melissa L. Rethlefsen, Matthew J. Page

**Affiliations:** 1 mlrethlefsen@gmail.com, Executive Director and Professor, Health Sciences Library & Informatics Center, University of New Mexico, Albuquerque, NM; 2 matthew.page@monash.edu, Senior Research Fellow and ARC DECRA Fellow, Methods in Evidence Synthesis Unit, School of Public Health and Preventive Medicine, Monash University, Melbourne, Australia

**Keywords:** PRISMA 2020, PRISMA-S, flow diagram, literature search, systematic reviews, reporting guidelines

## Abstract

The PRISMA 2020 and PRISMA-S guidelines help systematic review teams report their reviews clearly, transparently, and with sufficient detail to enable reproducibility. PRISMA 2020, an updated version of the PRISMA (Preferred Reporting Items for Systematic reviews and Meta-Analyses) statement, is complemented by PRISMA-S, an extension to PRISMA focusing on reporting the search components of systematic reviews. Several significant changes were implemented in PRISMA 2020 and PRISMA-S when compared with the original version of PRISMA in 2009, including the recommendation to report search strategies for *all* databases, registries, and websites that were searched. PRISMA-S also recommends reporting the number of records identified from each information source. One of the most challenging aspects of the new guidance from both documents has been changes to the flow diagram. In this article, we review some of the common questions about using the PRISMA 2020 flow diagram and tracking records through the systematic review process.

In early 2021, two reporting guidelines were released that provide direct guidance on how to report the literature search components of systematic reviews and related review types: PRISMA 2020, the updated version of the PRISMA (Preferred Reporting Items for Systematic reviews and Meta-Analyses) statement [1, 2]; and PRISMA-S, an extension to PRISMA focused solely on reporting the search components of systematic reviews [3]. PRISMA 2020 and PRISMA-S include several significant changes from the original version of PRISMA published in 2009 [4], including the recommendation to report search strategies for *all* databases, registries, and websites that were searched.

One of the most challenging aspects of integrating the new guidance from both documents into practice has been changes to the PRISMA flow diagram, which tracks the flow of information through the systematic review process. In the original version, the flow diagram was broken into four sections: identification, screening, eligibility, and included [4]. The identification section included boxes for recording the number of records identified through database searching, the number of records identified through other sources, and the number of records after deduplication. Often, using the PRISMA 2009 flow diagram, the “records identified through other sources” box contained only the number of records matching inclusion criteria, not necessarily all the records identified and screened. Building upon the original PRISMA 2009 flow diagram, PRISMA-S recommends constructing the flow diagram to show the number of records retrieved per database in the “records identified through database searching” box. Additionally, PRISMA-S asks authors to record the number of records retrieved for each other information source in the “records identified through other sources” box [3]. PRISMA-S also suggests reporting the total number of references retrieved from all sources, including updates, in the results section and the total number of references from each database and information source in the supplementary materials.

With the new PRISMA 2020 flow diagram template, systematic review teams now have the opportunity to better represent the complexity of the search process [1]. There are now four templates available, including flow diagram templates designed specifically for updates and systematic reviews that search beyond databases and study registries [5]. Generally, most systematic review teams will use the “PRISMA 2020 flow diagram for new systematic reviews which included searches of databases, registers and other sources” (see Figure 1 for an example) [5]. In this flow diagram, records are tracked through two different columns: identification of studies via databases and registers (Column 1) and identification of studies via other methods (Column 2). The flow diagram itself provides guidance on what type of information resource should be reported in which column, specifically noting that records identified from websites, organizations, citation searching, and other methods should be reported in Column 2. The flow diagram template also suggests reporting an overall number for records identified from databases and registers in Column 1.

**Figure 1:**
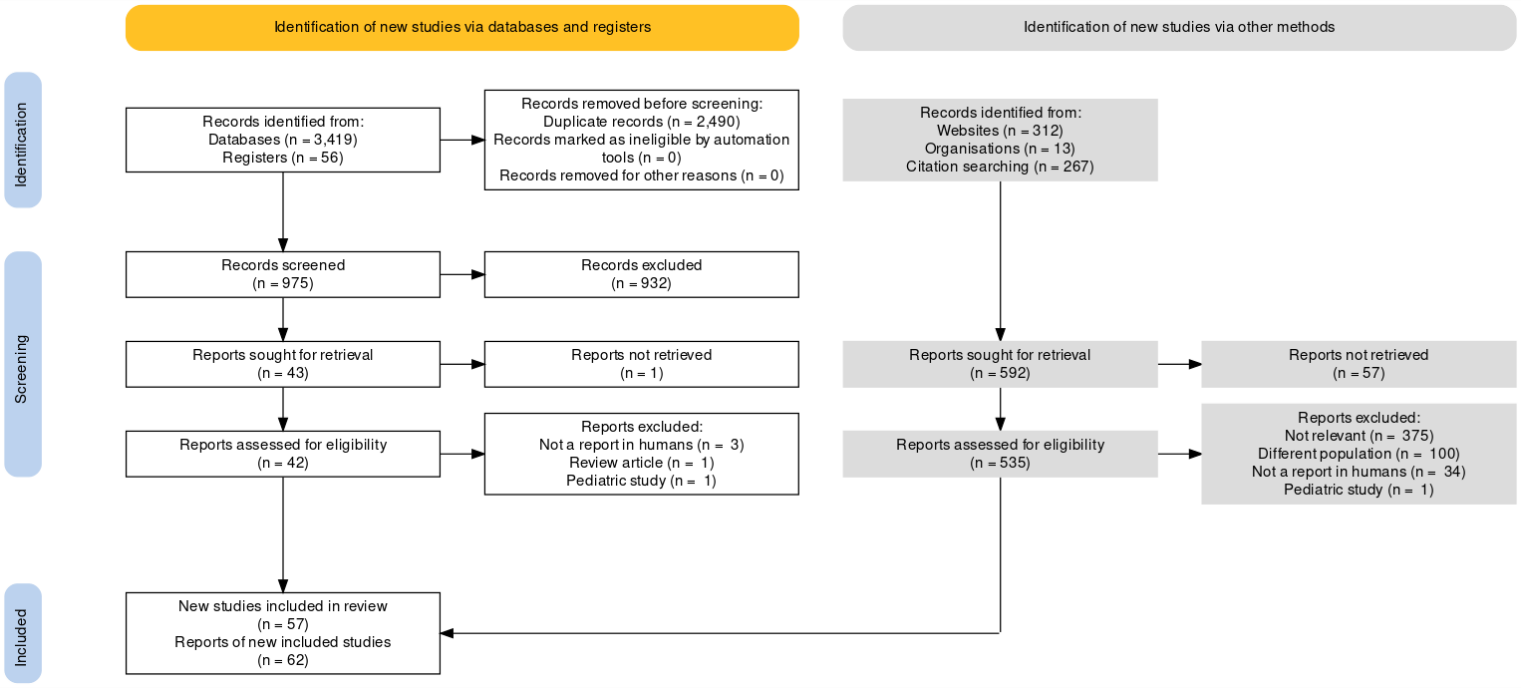
Example of a “PRISMA 2020 flow diagram for new systematic reviews which included searches of databases, registers and other sources” made using the R ShinyApp [1, 6, 7]

In the PRISMA 2020 flow diagram, Column 2 represents the “Additional records identified through other sources” from the PRISMA 2009 flow diagram but with major improvements to enhance tracking the entire flow of information through the systematic review process. Using the PRISMA 2009 flow diagram, many researchers only put the total number of records that met inclusion criteria in the “Additional records” box, thus excluding the total number of records that were retrieved from each source. The PRISMA 2020 flow diagram makes it explicit that it is expected that the total number of records retrieved from each information source should be tracked, which aligns with PRISMA-S's guidelines.

Since the publication of PRISMA 2020 and PRISMA-S, researchers have posed many questions about the best ways to track records and use the PRISMA 2020 flow diagram appropriately. In the rest of this commentary, we will answer some of the most common ones.

## Where can I access the PRISMA 2020 flow diagram?

All four versions of the PRISMA 2020 flow diagram are available in Word format on the PRISMA website [5]. In addition, there is a very useful R ShinyApp that creates downloadable flow diagrams from inputted data [6, 7].

## Do I need to seek permission from the authors to include a PRISMA 2020 flow diagram in my systematic review manuscript?

No, permission is not required. The PRISMA 2020 papers that include the flow diagram templates were published as open access articles distributed in accordance with the terms of the Creative Commons Attribution (CC BY 4.0) license, which permits others to distribute, remix, adapt, and build upon this work, even for commercial use, provided the original work is properly cited.

## PRISMA-S's flow diagram example matches the old PRISMA 2009 flow diagram. How should we comply with both PRISMA 2020 and PRISMA-S?

PRISMA-S was released slightly before PRISMA 2020, so the styles do not entirely align, but they are compatible—with a few tweaks. In PRISMA-S, the flow diagram example shows study registries data in the “Additional records identified through other sources” box (e.g., ClinicalTrials.gov) [3]. In the PRISMA 2020 flow diagram, Column 1 contains all data related to records and studies identified in study registries, and Column 2 now contains all records identified outside of databases and study registries, such as websites, reference lists, and contacts with manufacturers, among others [1].

PRISMA-S does recommend that, if space is available, individual databases and other information sources' identified records should be included in the flow diagram. This is not currently possible to do using the R ShinyApp [6], but any of the Word templates can be modified to add this information [5]. If it is not possible, the number of records per individual information source should go in the supplementary materials.

## The PRISMA 2020 flow diagrams puts “citation searching” in Column 2, but citation indexes are databases. If citation indexes are used to create lists of citing or cited references, which column should the records tracking data go in?

It may be helpful to put all citation searching results in Column 2 and reserve Column 1 for reporting subject-based searching, but it is not necessary to do so; users are free to modify the PRISMA 2020 flow diagram templates in a way they consider most optimal for their review. Citation indexes are indeed databases and can be included in Column 1, particularly if the records are assessed as part of the primary screening process [2]. As with all other searches, researchers conducting citation searches for citing or cited references should report the number of records identified per search in the supplementary materials. It is also important to cite each “base” article examined for citing or cited references in the manuscript text for reproducibility and transparency [3].

## It looks like the new PRISMA 2020 flow diagram wants us to list the number of records identified from other methods, like browsing reference lists, email alerts identifying citing articles, websites, contacts, etc. Is it really necessary to count all the records identified in these sources? Normally, we just report the items we identified that meet our inclusion criteria.

Identifying records and studies from other methods and information sources is one of the trickiest components of a systematic review to report. As acknowledged by the PRISMA 2020 flow diagram, those components of the review often take place outside the “normal” flow of screening that happens during the systematic review process. The ability (or lack thereof) to track the initial number of records identified is often determined by the process used to identify and screen the records—and the system used to manage records identified from other information sources. When records are all centrally tracked, regardless of source, it is easier to produce this data.

Best practice is to count all records identified (by hand or by other means) from each source. This information should be reported individually in supplementary materials, according to PRISMA-S [3]. It should also be reported in the PRISMA 2020 flow diagram [5], either by individual information source or by category of information source (i.e., all records identified from websites). If it is not possible or feasible to count all records, report what is feasible.

## Does Google Scholar count as a database or as an additional information source for the PRISMA 2020 flow diagram? What about Google?

Google Scholar is both a database and a citation index, and systematic review teams often use Google Scholar for both reasons. For subject-based searching, Google Scholar is considered as a database for the PRISMA 2020 flow diagram and can be reported in Column 1. If Google Scholar is only used as a citation index, data can be reported in either Column 1 or Column 2. The number of records identified should be reported per search in the supplementary materials, per PRISMA-S. If a systematic review team searches Google Scholar as both a traditional bibliographic database and as a citation index, the team may wish to use both Column 1 (subject-based search) and Column 2 (citation searching), but it is also reasonable to combine them in Column 1 in the flow diagram. Each search, however, needs to be reported separately in the supplementary materials.

Google, on the other hand, is not a traditional bibliographic database nor a citation index. It should be considered as an additional information source and reported in Column 2.

A complication of both Google and Google Scholar is that a maximum of 1,000 records is available for any given search, including citation searches [3]. Therefore, the total number of records identified from these two sources should never be listed in the flow diagram as above 1,000 for any given search. Many times, review teams will pre-identify how many records in Google or Google Scholar they will review per search; this should be the number reported for each search, unless the true number of results identified from a search is smaller.

## The PRISMA 2020 flow diagram R ShinyApp doesn't allow users to enter records by individual database, study registry, or other information source, like PRISMA-S recommends. Is that okay?

Yes. Though it is quite convenient to have that detail in the flow diagram, it is not essential to present it there. The number of records identified for each individual database and information source should be reported, however, in the supplementary materials regardless of whether they are included in the flow diagram. If a research team or publication prefers to report these records and sources in both places, the Word templates for the PRISMA 2020 flow diagram are customizable [5]. The R ShinyApp development team is also actively considering improvements so this feature may be available in future versions [6, 7].

## Before publishing our systematic review, we reran all the searches. What is best practice for PRISMA 2020 and PRISMA-S on reporting the number of identified records?

PRISMA-S treats all results from the same search, regardless of whether it was the original search or an update, as a single data point [3]. In the PRISMA 2020 flow diagram, report the total number of items retrieved per database across the lifespan of the systematic review searching process [5]. If multiple separate searches occurred for a particular database, the total results from each search can be combined in the flow diagram. Authors can consider reporting the number of records retrieved at each search point, original plus update(s), in the supplementary materials.

## What should be reported in the “Reports not retrieved” boxes in the PRISMA 2020 flow diagram?

There are occasions where reports cannot be located, for a multitude of reasons. This may include a journal that cannot be accessed in a local collection or via interlibrary loan, lack of response from authors or contacts, or broken links. Use the appropriate column's “Reports not retrieved” box to indicate how many reports were not able to be retrieved, regardless of reason.

## What is the distinction between the number of “Studies included in review” and “Reports of included studies,” which appears in the final box in the PRISMA 2020 flow diagram?

On some occasions, authors might identify a study that has results appearing in two reports (one providing data at three months, another at two years follow-up). In this case, the number of studies included in the review is one, whereas the number of reports of included studies is two. This distinction was introduced in the PRISMA 2020 flow diagram based on our observation that the jump from the number of *reports* assessed for eligibility to the number of *studies* included in the review (as was prompted in the original PRISMA flow diagram) sometimes resulted in some reports not being accounted for [2]. For example, we have seen some flow diagrams where the authors report assessing fifty full-text *reports* for eligibility, excluding forty *reports*, and including eight *studies* (failing to indicate that two of the eight studies were published in two reports).
